# Evaluation of cow's milk allergy

**DOI:** 10.1186/1939-4551-8-S1-A183

**Published:** 2015-04-08

**Authors:** Patricia Salles Cunha, Nathalia Pessoa Simis, Bruna Gama Saliba, Ariana Yang, Milene Yamashita, Jorge Kalil, Fabio Fernandes Morato Castro

**Affiliations:** 1 University of São Paulo, Brazil

## Background

The prevalence of food allergy (FA) is increasing; consequently the number of patients on elimination diet (ED) due to a suspected diagnosis is also increasing. Diagnosis of food allergy should be based on a convincing history of allergic reactions or on the result of an oral food challenge test. The oral food challenge test (OFC) is the most reliable clinical procedure for diagnosing food allergy.

## Methods

The goal of this study was evaluated the proportion of individuals that were following an elimination diet unnecessarily, because of either acquired tolerance or misdiagnosis. A retrospective analysis of patient’s records was made. We evaluated patients that were following elimination diet of cow´s milk. They underwent oral challenge with cow´s milk. The oral food challenge was carried out for two purposes: to confirm the diagnosis of cow's milk allergy or to demonstrate development of clinical tolerance to allergic food. The oral food challenge was performed like open test and double bind placebo controlled.

## Results

The oral challenge to cow´s milk were performed in 33 patients with a mean age of 17 years old (1-86 yo). In this series 63.6% patients were female. Atopic diseases were recorded in 45% of patients (15/33). Among the OFC, 20 were performed to confirm diagnosis of cow´s milk allergy; and 13 to demonstrate development of clinical tolerance in patients previously allergic. We found 27/33 (81%) negative OFC. The food challenge test confirmed food allergy to cow milk only in 4/20 patients (20%) who had suspected milk allergy and were following elimination diet. The positive results in specific IgE and in skin prick tests were recorded in 48.1% of patients. According to the open exposure tests and double-blind, placebo controlled food challenge tests these patients are only sensitized to cow's milk without clinical symptoms of allergy. Among the patients allergic to cow's milk 91% (11/12) were already tolerant to milk.

## Conclusions

The mean age of patients was high. Most of them were following an elimination diet unnecessary, since they did not have cow´s milk allergy or they were tolerant. The lack of specialized services to perform oral challenge tests slows diagnostic confirmation.

